# P53 Function Status Correlates With Overall Survival in Patients With Resected Pancreatic Cancer

**DOI:** 10.1002/jso.70060

**Published:** 2025-08-07

**Authors:** Hui Chen, Alisha Agarwal, Sreekanth Yellanki, Harish Lavu, Richard Zheng, Wilbur B. Bowne, Charles J. Yeo, Aditi Jain, Avinoam Nevler

**Affiliations:** ^1^ Jefferson Pancreas, Biliary, and Related Cancer Center, Department of Surgery Thomas Jefferson University Philadelphia Pennsylvania USA

**Keywords:** gain‐of‐function, oncologic outcomes, P53, pancreatic ductal adenocarcinoma, PDAC

## Abstract

**Background:**

The P53 gene is the most common tumor‐suppressor gene mutated in pancreatic ductal adenocarcinoma (PDAC). The gene's normal function is critical for regulation of replication, DNA repair, and apoptosis. The purpose of our study is to determine the impact of the various P53 mutation subtypes on survival in resected PDAC.

**Methods:**

This is a retrospective cohort study assessing patients that underwent curative‐intent resection for PDAC between the years of 2016–2022. Next generation sequencing (NGS) was performed on patient tumors. P53 tumor genotypes were grouped into wild‐type (WT), gain‐of‐function (GOF) mutations (R175H, R248W, R248Q, R273H, R282W, G245S) and all other non‐GOF mutations.

**Results:**

The study included a total of 330 patients with resected PDAC. P53 mutations were found in tumors of 243 patients (74%), and 87 (26%) patients had WT P53. Among patients with mutant P53 tumors, 58 patients (24%) had a GOF mutation, and 185 patients (76%) had a non‐GOF mutation. Survival analysis showed that non‐GOF P53 mutations were associated with the shortest overall survival compared with WT and GOF (25.6 ± 2.4 months vs. 32.2 ± 3.6 months, vs. 36.2 ± 4.4 months, respectively. *p* = 0.038). Similarly, non‐GOF mutations were associated with the shortest disease‐free survival (14.6 ± 1.2 months, vs. 19.6 ± 3.5 months, vs. 18.3 ± 3.6 months, respectively. *p* = 0.039).

**Conclusions:**

Our data suggest that P53 mutations grouped by functional status may hold differential prognostic value regarding survival and recurrence of patients with PDAC. Further investigations are required to validate these findings.

## 1 Introduction

Pancreatic ductal adenocarcinoma (PDAC) is a highly aggressive gastrointestinal malignancy which has become, over the past decade, the third leading cause of cancer‐related mortality in the United States [1]. Despite advances in oncologic and surgical therapy, survival remains poor, with 5‐year overall survival rates of only 12%–13% [2, 3]. The recent advances and access to molecular sequencing have allowed a deeper investigation into the heterogeneity of PDAC gene mutations and their impact on the course of the disease.

P53 mutations occur in 50%–70% of all PDAC cases [4, 5]. P53 is the most frequently altered gene in human tumors [6], and it plays a critical role in regulating cell growth, DNA repair, and apoptosis. It imparts cellular resilience against various environmental stressors such as DNA damage, oxidative stress, nutrient deprivation, and antitumor immune responses [7]. Notably, recent reports suggest that P53 mutation subtypes in PDAC are associated with distinct cellular phenotypes which may have prognostic implications [8, 9]. These studies suggest that gain‐of‐function (GOF) mutations in P53 can be linked to poorer overall survival rates in PDAC compared to non‐GOF mutations [8, 9]. In vitro studies of PDAC cells link certain GOF mutations such as R172H with inhibition enzymes in urea metabolism, attenuating tumorigenic metabolism [10]. However, other studies suggest that a deficiency or loss of function (LOF) in P53 is itself linked to a poorer survival in PDAC [11]. For example, the non‐GOF mutation R270H in PDAC cells was shown to inhibit glutathione synthesis and reduction of oxidative stress in the mitochondria [10]. In ovarian cancer, Madeddu et al. [12] have shown that P53 LOF was associated with worse survival outcomes and reduced response to platinum therapy.

In this study, we aim to assess the prognostic significance of P53 mutant subtypes in our cohort of patients with PDAC who underwent curative‐intent resection.

## 2 Methods

### 2.1 Study Population

This was a retrospective cohort study of all patients undergoing curative‐intent pancreatic resection for PDAC at the Thomas Jefferson University Hospital between the years of 2016–2022. Patients with PDAC who received curative‐intent surgery and had next generation sequencing (NGS) data were identified and screened for completeness of clinical data. This study was performed using data from a prospectively maintained Thomas Jefferson University Hospital Pancreatic Cancer database as well as individual patient electronic medical records.

### 2.2 Data Collection

Patient demographic, tumor characteristics, perioperative care, surgical and oncological outcome data were recorded for all patients in this study. Resected pancreatic specimens were analyzed for somatic mutations using an in‐house NGS panel for detecting hot‐spot region mutations in 42 cancer‐related genes, as previously described [13]. Formalin‐fixed, paraffin‐embedded (FFPE) tumor tissue slides were reviewed by a pathologist and marked for tumor containing regions. Subsequently, cancer DNA was extracted from the corresponding FFPE tissue and processed using the Illumina TruSeq Amplicon Cancer Panel.

P53 tumor genotypes were grouped into wild‐type (WT), GOF mutations, and non‐GOF mutations. P53 GOF mutations included were R175H, R248W, R248Q, R273H, R282W, and G245S, as described by previous literature [14]. Non‐GOF mutations included all other P53 mutations observed, including loss of function (LOF) mutations. As the definition of LOF mutations can vary between studies, we opted to classify our P53 mutations as GOF and non‐GOF.

### 2.3 Statistical Analysis

Statistical analyzes were conducted using SPSS, version 29.0.1.0 (IBM Corp, Armonk, NY) software. Disease‐free survival (DFS) and overall survival (OS) of the P53 groups were compared using a Kaplan‐Meier analysis and Log‐Rank comparison. Confidence intervals (CI) reported were 95%. *p* values < 0.05 were considered as statistically significant. A Cox regression model was used to assess and quantify independent prognostic factors for DFS and OS. The factors examined included age at surgery, neoadjuvant chemotherapy, adjuvant chemotherapy, T‐stage, N‐stage, perineural invasion and lymphovascular invasion. In the multivariate Cox regression analysis, variable selection was guided by a stepwise backward elimination process. The model was initiated with all potential predictor variables and iteratively removed the least significant variable until no improvement in Akaike Information Criterion (AIC) or *p*‐value.

### 2.4 Ethical Approval

This study was conducted in accordance with the ethical standards of the Institutional Review Board at Thomas Jefferson University Hospital (IRB#22E.136). The data supporting the findings of this study are available upon request.

## 3 Results

### 3.1 Cohort Characteristics

The study included a total of 330 patients with resected PDAC, as detailed in Table 1. The mean age was 68.6 ± 8.8 years, and the male to female ratio was 168:162. P53 mutations were found in 243 patients (74%), and 87 (26%) patients had WT P53. Of the patients with mutant P53 tumors, 58 patients (24%) had a gain of function (GOF) mutation, and 185 patients (76%) had a non‐GOF mutation. The most common P53 mutation was the GOF mutation R175H, observed in 16/243 patients (7%) of all PDAC patients with mutations. Other common GOF mutations included R273H in 10 patients (4%), R248W in 10 patients (4%), and R248Q in 7 patients (3%). Some of the common non‐GOF mutations include R273C in 11 patients (5%), Y220C in 11 patients (5%), and G245S in 8 patients (3%).

**Table 1:** Patient characteristics, grouped by P53 mutational subgroup (wild‐type, GOF, non‐GOF).

Mean (± SD)/*n* (%)	Overall (*n* = 330)	Wildtype (*n* = 87)	GOF (*n* = 58)	non‐GOF (*n* = 185)	Sig.
Age, year	68.6 (8.8)	69.3 (8.9)	68.6 (8.1)	68.4 (9.0)	0.75
Sex					0.4
Female	162 (49.1)	40 (46.0)	33 (56.9)	89 (48.1)	
Male	168 (50.9)	47 (54.0)	25 (43.1)	96 (51.9)	
Race					0.94
White/Caucasian	270 (81.8)	73 (83.9)	49 (84.5)	148 (80.0)	
Black/African American	30 (9.1)	7 (8.1)	3 (5.2)	20 (10.8)	
Asian	15 (4.5)	3 (3.5)	3 (5.2)	9 (4.9)	
Hispanic/Latinx	12 (3.6)	3 (3.5)	2 (3.5)	7 (3.8)	
Other	3 (1.0)	1 (1.0)	1 (1.6)	1 (0.5)	
Neoadjuvant Chemo	89 (27.0)	24 (27.6)	14 (24.1)	51 (27.6)	0.87
Adjuvant Chemo	264 (79.3)	62 (69.7)	51 (86.4)	151 (81.6)	0.03
Tumor size (cm)	3.0 (1.3)	2.9 (1.5)	2.7 (1.2)	3.1 (1.2)	0.2
Positive margins	39 (11.7)	11 (12.4)	6 (10.2)	22 (11.9)	0.91
Lymphovascular	164 (49.7)	32 (36.8)	25 (43.1)	107 (57.8)	0.01
Perineural invasion	294 (89.1)	72 (82.8)	52 (89.7)	170 (91.9)	0.08
Tumor staging					0.2
T1	60 (18.2)	17 (19.5)	15 (25.9)	28 (15.1)	
T2	169 (51.2)	40 (46.0)	32 (55.1)	97 (52.4)	
T3	96 (29.0)	30 (34.5)	10 (17.3)	56 (30.3)	
T4	3 (1.0)	0 (0)	1 (1.7)	2 (1.1)	
TX	2 (0.6)	0 (0)	0 (0)	2 (1.1)	
Nodal staging					0.37
N0	114 (34.5)	35 (40.4)	22 (37.3)	57 (30.8)	
N1	129 (39.2)	34 (39.1)	22 (37.9)	73 (39.5)	
N2	86 (26.3)	17 (19.5)	14 (23.7)	55 (29.7)	

*Note:* GOF, gain‐of‐function; Sig, significance.

Patients in the non‐GOF mutation group displayed higher rates of lymphovascular invasion (57.8%) compared to both GOF (43.1%) and WT (36.8%; *p* = 0.01) tumors. Both the GOF and the non‐GOF mutation groups had higher rates of adjuvant chemotherapy administered compared to WT group (86.4% for GOF and 81.6% for non‐GOF vs. 69.7% for WT, *p* = 0.03). Other characteristics between cohorts were fairly similar. Radiation therapy was used in 73 of 330 patients (22.1%). Thirteen out of 87 (14.9%) WT patients, 16 out of 58 (27.6%) GOF patients, and 44 out of 185 (23.8%) non‐GOF patients received radiation (*p* = 0.14). The main chemotherapy regimens used were either FOLFIRINOX or Gemcitabine‐based. Twenty‐one patients had exposure to both regimens. FOLFIRINOX was used in 125 of 330 (37.9%) patients. 26 of 87 (29.9%) WT patients, 25 of 58 (43.1%) GOF patients, and 74 of 185 (40.0%) non‐GOF patients received FOLFIRINOX (*p* = 0.18). Gemcitabine‐based therapy was used in 142 of 330 (43.0%) patients. Forty‐six of 87 (52.9%) WT patients, 23 of 58 (39.7%) GOF patients, and 73 of 185 (39.5%) non‐GOF patients received Gemcitabine‐based therapy (*p* = 0.10). Of note, the GOF group tended to present with tumors of an earlier T‐stage (25.9% T1), although that finding was not statistically significant.

### 3.2 Survival Analysis

**Figure 1:**
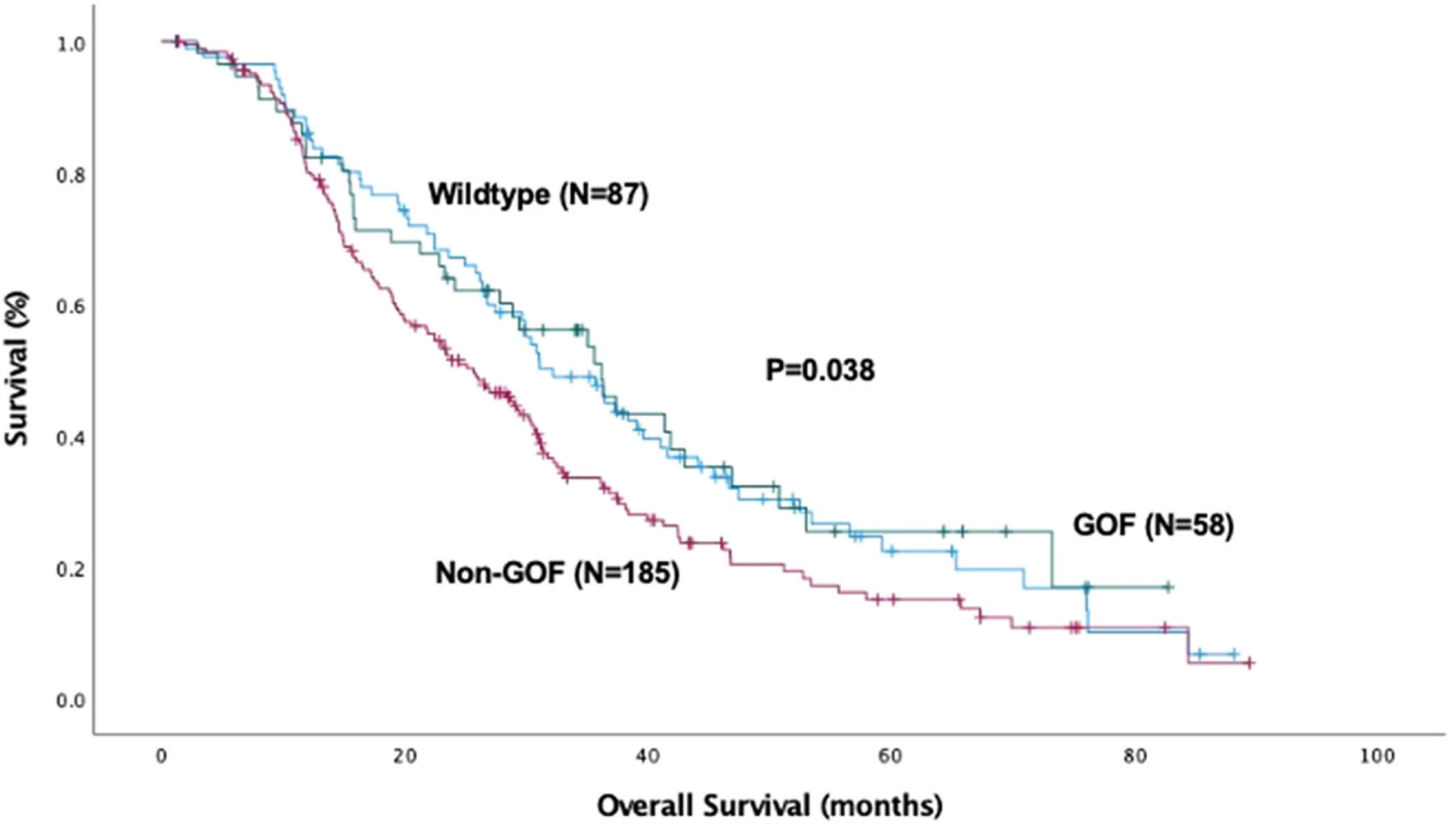
Kaplan‐Meier curve for overall survival (OS) by P53 mutation type. The median overall OS for the cohort was 29.4 ± 1.4 months (OS_Wild‐Type_: 32.2 ± 3.6 months, OS_GOF_: 36.2 ± 4.4 months, and OS_non‐GOF_: 25.6 ± 2.4 months, *p* = 0.038). GOF, gain‐of‐function.

The median overall survival for the entire cohort was 29.4 ± 1.4 months and the disease‐free survival was 16.3 ± 0.83 months. Patients with non‐GOF mutations had the shortest overall survival of 25.6 ± 2.4 months, compared to WT patients (32.2 ± 3.6 months, *p* = 0.040) and GOF patients (36.2 ± 4.4 months, *p* = 0.049) as shown in Figure 1.

**Figure 2:**
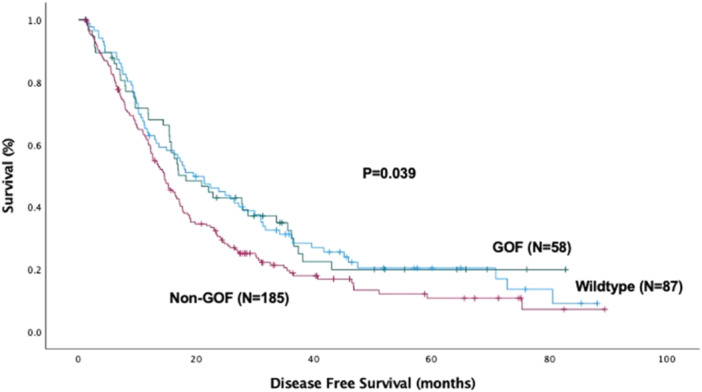
Kaplan‐Meier curve for disease‐free survival (DFS) by P53 mutation type. The median DFS for the entire cohort was 16.3 ± 0.83 months (DFS_Wild‐Type_: 19.6 ± 3.5 months, DFS_GOF_: 18.3 ± 3.6 months, and DFS_non‐GOF_: 14.6 ± 1.2 months, *p* = 0.039). GOF, gain‐of‐function.

A similar pattern was shown in the Kaplan‐Meier comparison of disease‐free survival: the non‐GOF group had the worst survival (14.6 ± 1.2 months) compared to WT (19.6 ± 3.5 months, *p* = 0.038) and, with a similar trend, GOF patients (18.3 ± 3.6 months. *p* = 0.051), as shown in Figure 2.

**Figure 3:**
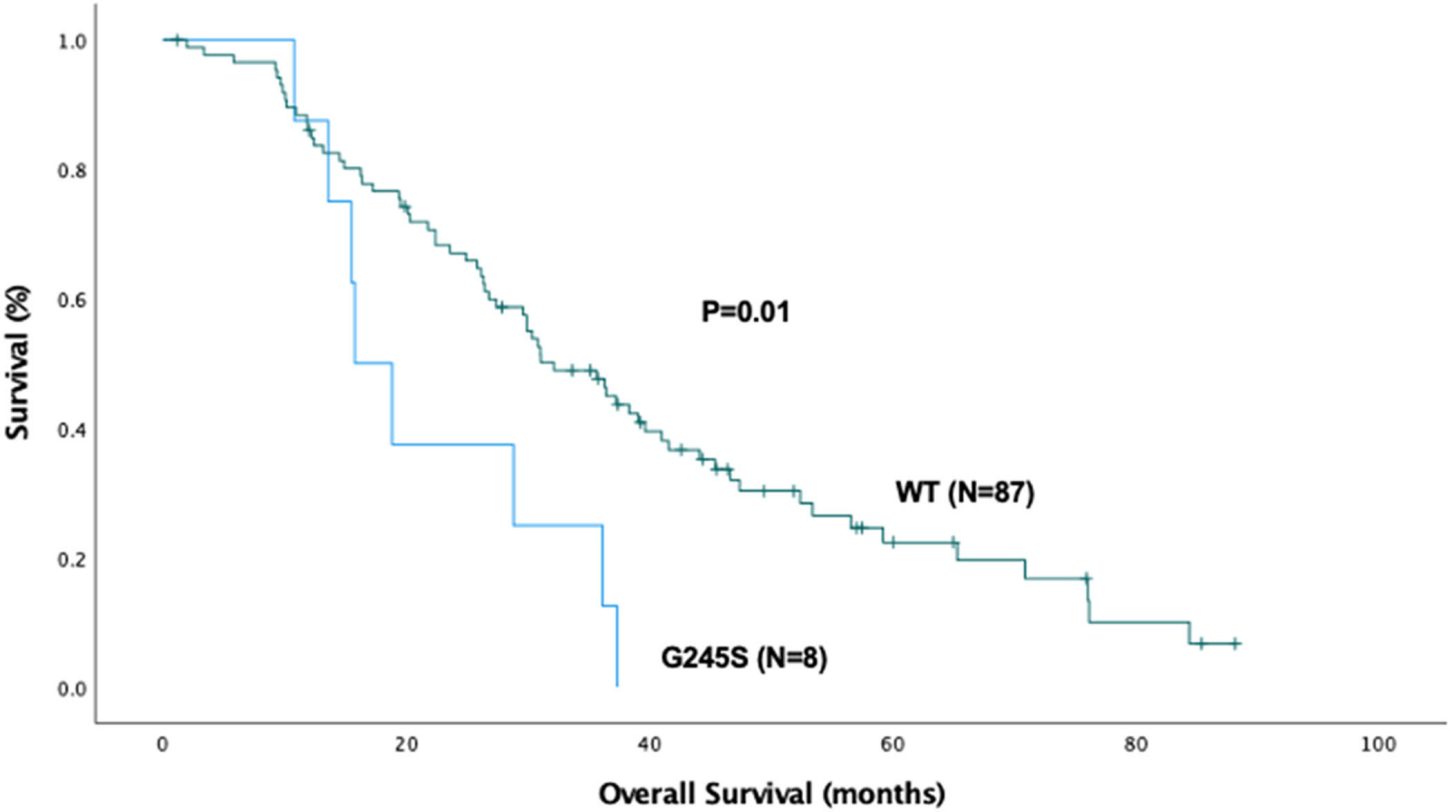
Kaplan‐Meier curve for overall survival (OS) comparing the wild‐type (WT) (*N* = 87) vs. P53 mutation G245S (*N* = 8). The median OS for the WT cohort was 32.2 ± 3.6 months and was 15.8 ± 2.4 months for the G245S group (*p* = 0.01).

**Figure 4:**
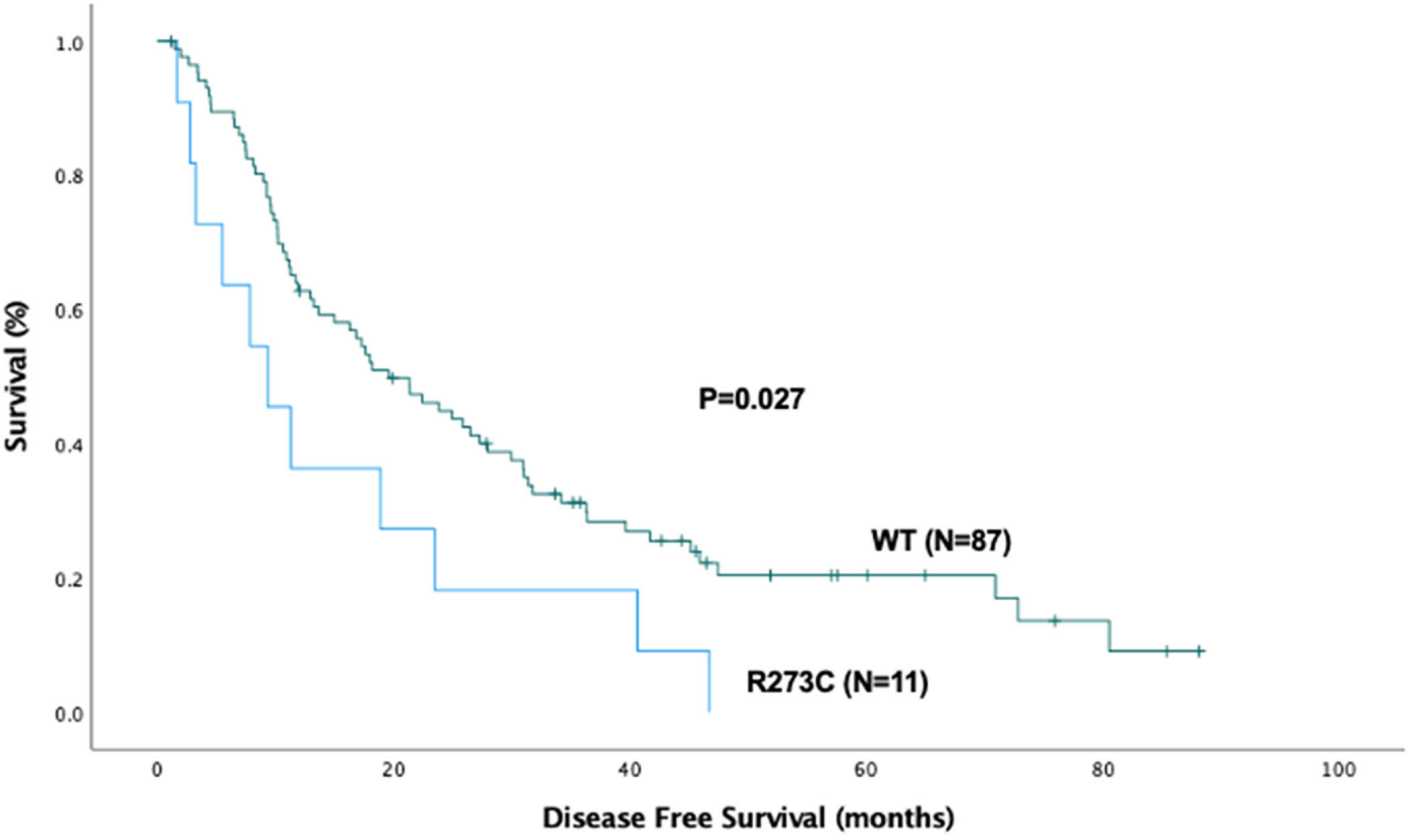
Kaplan‐Meier curve for disease‐free survival (DFS) comparing the wild‐type (WT) (*N* = 87) vs P53 mutation R273C (*N* = 11). The median DFS for the WT cohort was 19.6 ± 3.5 months and was 9.3 ± 3.2 months for the R273C group (*p* = 0.027).

When individual mutations were compared to WT, two mutation subtypes showed significant differences as shown in Figures 3 and 4. In the Kaplan‐Meier comparison of overall survival, the G245S mutation cohort (*N* = 8) had a worse median survival than WT (15.8 ± 2.4 months vs. 32.2 ± 3.6 months, respectively. *p* = 0.01). In a similar analysis for disease‐free survival, the R273C mutation cohort (*N* = 11) had a worse median disease‐free survival than WT (9.3 ± 3.2 months vs. 19.6 ± 3.5 months, respectively. *p* = 0.027).

In a subgroup analysis of patients who received FOLFIRINOX, patients with non‐GOF mutations had a lower overall median survival than patients with WT or GOF mutations (23.9 ± 3.3 months vs. 44.1 ± 8.7 months vs. 37.4 ± 6.3 months, respectively, *p* = 0.008), see Supporting information Figure 1. Similarly, patients with non‐GOF mutations had a lower disease‐free median survival than patients with WT or GOF mutations (12.7 ± 1.5 months vs. 22.4 ± 4.2 months vs. 18.3 ± 4.9 months, respectively, *p* = 0.05), see Supporting information Figure 2. Interestingly, in patients who received Gemcitabine‐based therapy, there was no significant difference in a Kaplan‐Meier comparison of overall median survival for patients when comparing mutational status (WT 27.4 ± 2.8 months vs. GOF 35.0 ± 10.8 months vs. non‐GOF 28.9 ± 4.2 months, *p* = 0.55), see Supporting information Figure 3. Similarly, there was no difference in a Kaplan‐Meier comparison of disease‐free median survival when comparing mutational status (WT 11.7 ± 1.5 months vs. GOF 16.9 ± 2.2 months vs. non‐GOF 14.6 ± 1.6 months, *p* = 0.44), see Supporting information Figure 4.

Assessment of radiotherapy response reveals that there was no significant difference when comparing mutational status for either overall survival (*p* = 0.73) or disease‐free survival (*p* = 0.58).

### 3.3 Impact of P53 Non‐GOF Mutation on Tumor Progression

Overall, the WT and GOF group had seemingly superior survival outcomes compared to the non‐GOF group, and these effects appeared to be mostly limited to the FOLFIRINOX treated patients. As these findings were in line with previously published data, suggesting increased platinum resistance in patients with non‐GOF mutant P53, we continued with an ad‐hoc two arm analysis, comparing patients with non‐GOF tumors to the rest of the cohort. This was also beneficial to increase statistical power for the comparisons, thereby creating a non‐GOF group (*N* = 185) and WT/GOF group (*N* = 145).

**Figure 5:**
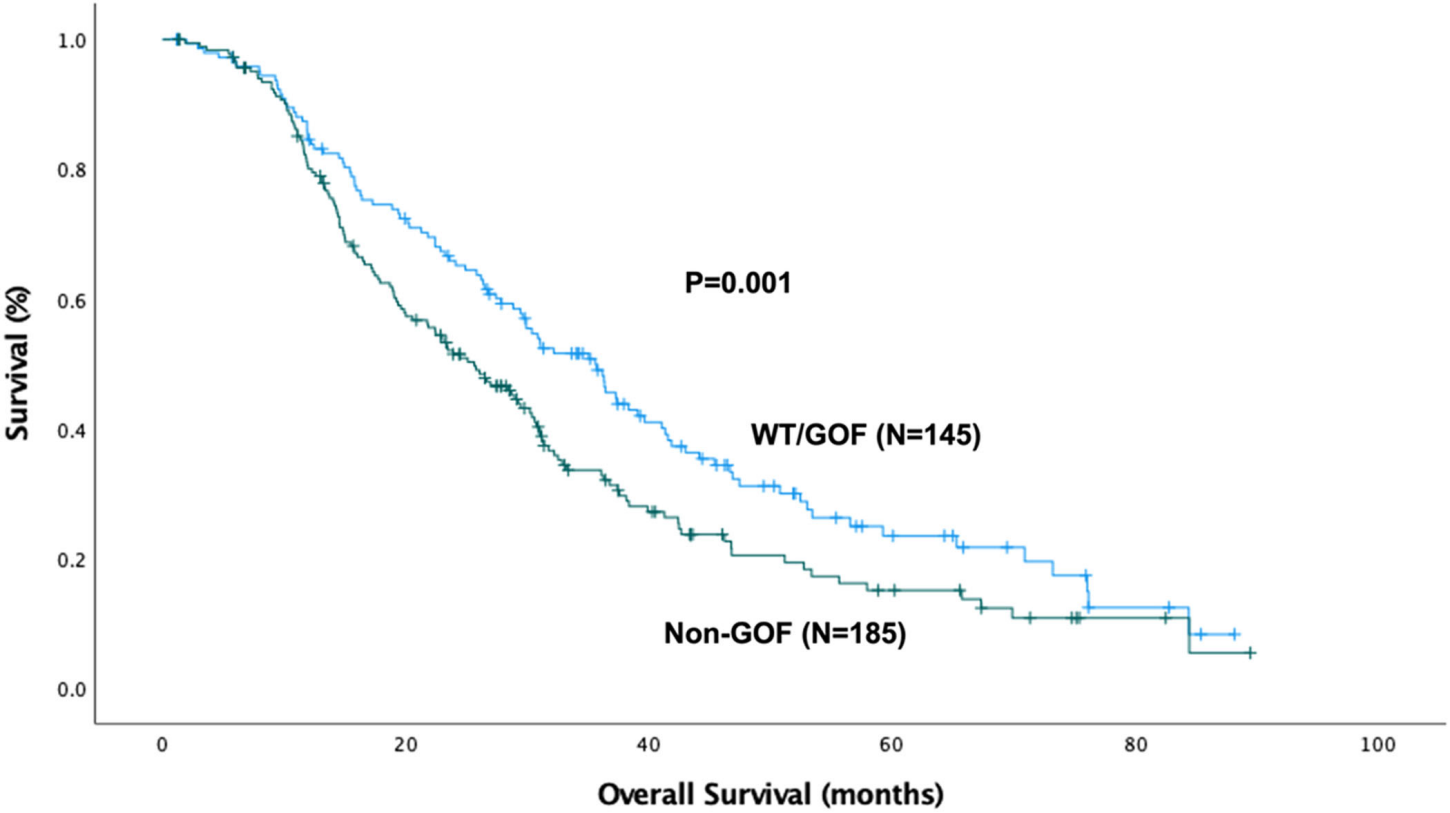
Kaplan‐Meier curve for overall survival (OS) comparing the wild‐type (WT) and GOF groups (*N* = 145) vs. non‐GOF (*N* = 185). The median OS for the WT and GOF cohort was 35.6 ± 2.9 months and was 25.6 ± 2.4 months for the non‐GOF group (*p* = 0.01). GOF, gain‐of‐function.

**Figure 6:**
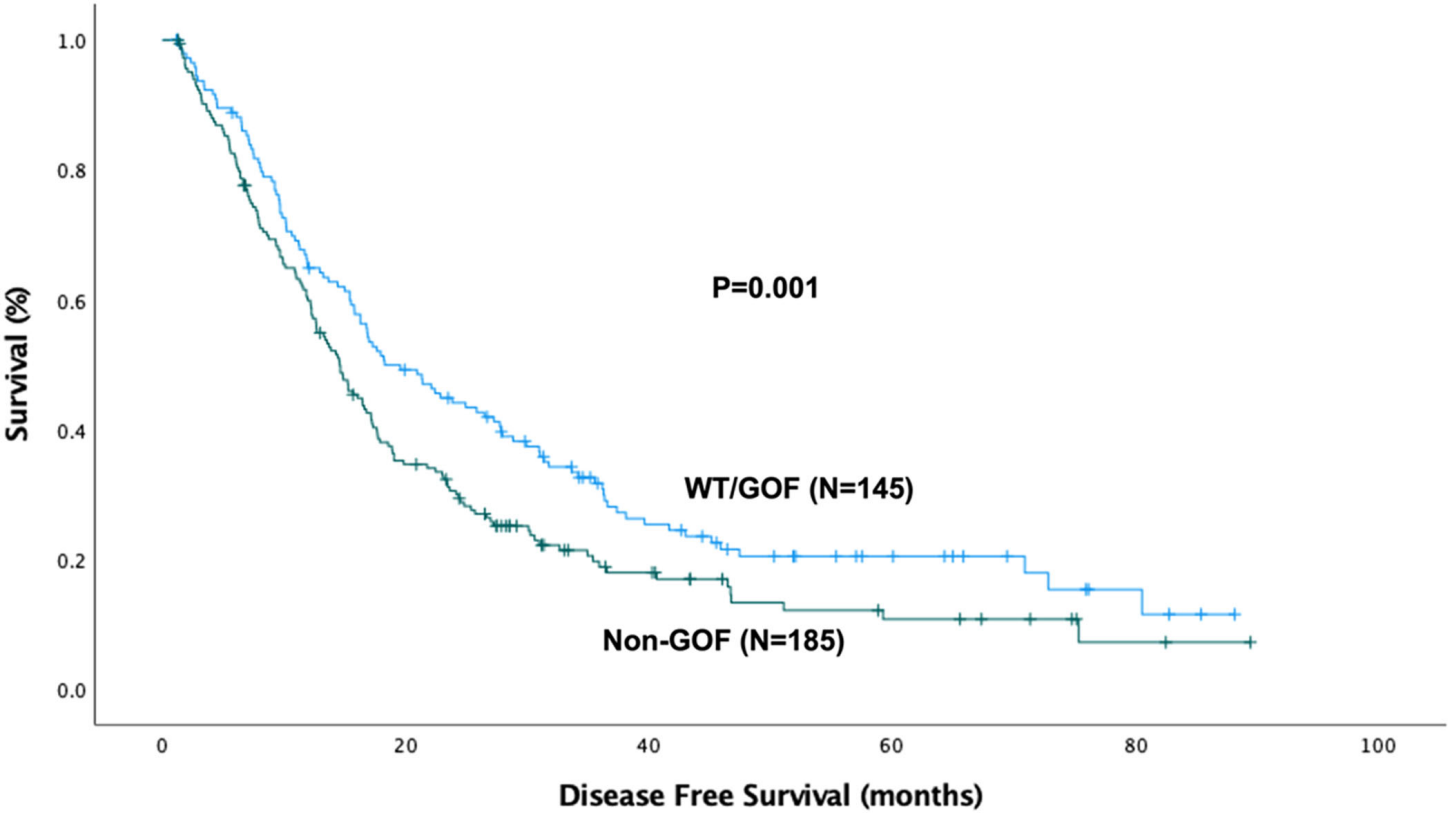
Kaplan‐Meier curve for disease‐free survival (DFS) comparing the wild‐type (WT) and GOF groups (*N* = 145) vs. non‐GOF (*N* = 185). The median DFS for the WT and GOF cohort was 18.3 ± 2.4 months and was 14.6 ± 1.2 months for the non‐GOF group (*p* = 0.01). GOF, gain‐of‐function.

Subsequent Kaplan‐Meier analyzes showed that non‐GOF patients had a shorter overall (25.6 ± 2.4 months vs. 35.6 ± 2.9 months, *p* = 0.01) and disease‐free survival (14.6 ± 1.2 months, vs. 18.3 ± 2.4 months, *p* = 0.01) compared to the combined WT/GOF group, as seen in Figures 5 and 6.

In a Cox‐regression analysis of overall survival, non‐GOF status was slightly more correlated with mortality compared to the WT/GOF combined group (HR 1.3, *p* = 0.05) as seen in Table 2. Tumor factors that correlate with increased mortality include lymphovascular invasion (HR 1.8, *p* < 0.001), perineural invasion (HR 1.8, *p* = 0.03), and positive margins (HR 1.8, *p* = 0.001). Adjuvant chemotherapy was a protective factor for survival (HR 0.59, *p* = 0.002).

**Table 2:** Optimized multivariable cox regression model of overall survival in patients after curative‐intent resection of pancreatic ductal adenocarcinoma.

Covariate	Hazard ratios [95% CI]	Sig.
Non‐GOF vs. WT/GOF	1.3 [1.0–1.7]	0.05
Adjuvant chemotherapy	0.59 [0.4–0.8]	0.002
Lymphovascular invasion	1.8 [1.3–2.4]	< 0.001
Perineural invasion	1.8 [1.0–2.9]	0.03
Positive margins	1.8 [1.3–2.6]	0.001

*Note:* GOF, gain‐of‐function; Sig, significance.

Cox‐regression analysis of disease‐free survival comparing non‐GOF status with WT/GOF patients revealed a higher hazard ratio for events, although this was not found to reach statistical significance (HR 1.3, *p* = 0.07), as seen in Table 3. Other factors correlated with increased morbidity were the requirement of neoadjuvant chemotherapy (HR 1.4, *p* = 0.02), lymphovascular invasion (HR 1.4, *p* = 0.01), perineural invasion (HR 1.8, *p* = 0.02), and positive margins (HR 1.5, *p* = 0.04).

**Table 3:** Optimized multivariable cox regression model of disease‐free survival in patients after curative‐intent resection of pancreatic ductal adenocarcinoma.

Covariates	Hazard ratios [95% CI]	Sig.
Non‐GOF vs. WT/GOF	1.3 [0.98–1.7]	0.07
Neoadjuvant chemotherapy	1.4 [1.1–1.8]	0.02
Adjuvant chemotherapy	0.76 [0.6–1.0]	0.09
Lymphovascular invasion	1.4 [1.1–1.9]	0.01
Perineural invasion	1.8 [1.1–3.0]	0.02
Positive margins	1.5 [1.0–2.1]	0.04
T stage	1.2 [0.97–1.4]	0.1

*Note:* GOF, gain‐of‐function; Sig, significance.

In a FOLFIRINOX‐specific subset of patients, Cox‐regression revealed the non‐GOF status correlated with poor overall survival (HR 2.0, *p* = 0.008) and poor disease‐free survival (HR 1.7, *p* = 0.016) compared to the WT/GOF subtypes group, see Supporting information Tables 1 and 2.

In a Gemcitabine‐based therapy subset of patients, Cox‐regression failed to uncover significant impact of P53 mutational subtype on overall survival (HR 0.87, *p* = 0.47), see Supporting information Table 3.

## 4 Discussion

PDAC is an aggressive and insidious disease with only modest improvements in its overall survival being observed over the past decades [3]. Several prognostic factors of survival have been described for PDAC including tumor grade and stage, lymphovascular and perineural invasion, margin status, completion of adjuvant therapy, and serum levels of CA19‐9 and CEA. With the advancement of technology and next‐generation sequencing, greater emphasis has been put into assessment of molecular risk factors to develop personalized management of PDAC. Our study aims to clarify the impact of P53 mutation subtypes on oncological outcomes in patients with early stage resected PDAC.

P53 is a tumor suppressor that controls senescence and apoptosis in response to oxidative and metabolic stress [15]. Additionally, specific P53 missense mutations can even result in activation of oncogenic properties [16]. P53 mutations can be grouped into two functional groups: LOF mutations and GOF mutations. A LOF mutation results in the inactivation of P53's tumor suppressing abilities, for example regulation of cell death in response to cellular stress. A GOF is indicated by the further acquisition of an additional oncogenic ability, which can present as increased invasiveness, decreased responsiveness to chemotherapy, increased proliferation, and changing the tumor microenvironment to promote oncogenesis [15]. Approximately 70% of our patients had P53 mutations, of which the majority (76%) had a non‐GOF mutation, which is concordant with prior published literature [9, 17, 18]. Our study classified P53 GOF mutations based on previously reported P53 missense mutations [19]. As the definition of LOF mutations can vary between studies, we opted to classify our P53 mutations as GOF and non‐GOF.

In light of multiple studies that show differential effects of P53 mutations across different cancer types, the effects of GOF and LOF P53 mutations appear to be tumor and cell‐line dependent, which may explain the somewhat inconsistent and, at‐times, conflicting reports. Klemke et al. reported that the P53 R248W GOF mutation specifically is associated with increased cell migration in PDAC [20]. Similarly, Morton et al. showed that the R172H P53 GOF mutation was associated with increased tumor cell invasion [21], and Efe et al. reported that the R172H P53 GOF mutation was associated with increased lung metastasis in esophageal squamous cell carcinoma models [22]. A retrospective cohort study of metastatic and locally advanced PDAC further revealed that GOF mutations increased the mortality rate by 27% (HR 1.27, 1.02–1.59 CI 95%) compared to non‐GOF mutations [8].

In contrast to these observations, Wang et al. have shown evidence in multiple cancer cell lines suggesting that the tumorigenic properties in P53 mutations stem from the LOF element of the mutation, rather than the GOF [23]. That hypothesis is also supported by the work of Wörmann et al. showing LOF in P53 was associated with aggressive features in‐vivo and poorer survival in PDAC patients [24]. Additionally, in resected high‐grade serous ovarian cancer, LOF P53 mutations had worse disease free survival compared to wildtype or GOF mutations [12]. Caporali et al. described a possible mechanism for the aggressive features of the non‐GOF p53 mutation R270H in mouse PDAC cells stemming from alterations to glutathione production and oxidative stress reduction in the mitochondria [10]. This aligns well with our data demonstrating that non‐GOF mutational status has a poorer overall survival with FOLFIRINOX‐based chemotherapy compared to WT or GOF survival. Additionally, Gemcitabine‐based chemotherapy susceptibility did not change based on mutational status, but the overall survival was lower when compared to FOLFIRINOX.

In our Kaplan‐Meier analyzes, we found that the G245S mutant patients cohort had worse overall survival compared to wild‐type P53, and the R273C mutant patients had worse disease‐free survival. Unfortunately, the small size of the numerous subtype patient populations, resulted in lack of statistical power to detect other differences. P53 G245S is a GOF mutation that destabilizes the DNA binding domain and causes increased proliferation, metastasis, and invasion in esophageal squamous cell carcinoma [25]. A population study by Samowitz et al. demonstrated G245 mutations as one of the primary predictors of poor prognosis (HRR 2.16, 95% CI 1.06–4.40) [26]. Similarly, P53 R273C has been suggested in some studies to decrease DNA binding and impact cell proliferation and decreased tumor‐free survival in murine models of prostate cancer and in vitro studies of lung and breast cancer cell lines [27, 28].

Several studies have attempted to clarify the effect of P53 mutational subtype on tumor progression. A recent study by Pan and colleagues assessed a retrospective cohort of metastatic and locally advanced PDAC [8]. In the study, P53 mutations were grouped into GOF and non‐GOF groups, which revealed that GOF mutations were associated with a worse prognosis compared to non‐GOF [8]. Interestingly, our study in resected PDAC has found an opposite pattern; the GOF group was characterized by tumors with less lymphovascular invasion, resembling the P53 WT group, and non‐GOF group had a shorter overall and disease‐free survival compared to WT and GOF groups.

While prior studies centered on locally advanced PDAC, our study focused on resected PDAC, which may have uncovered a point of differentiation. Our own retrospective cohort study on patients with advanced PDAC receiving at least one line of therapy, Zohar et al. found that P53 WT patients had a longer median overall survival (2.1 years [1.8–2.4, 95% CI]) than patients with a GOF (1.5 years [1.3–2.1, 95% CI]) or non‐GOF (1.4 years [1.3–1.5, 95% CI]) mutations [29]. A higher co‐occurrence of KRAS and ATM mutations in patients with P53 mutations compared to WT was also found in this study, which may play a role in PDAC progression [29].

There are several limitations to our study which merit further discussion to clarify interpretations of the results. One limitation of our study includes grouping mutational subtypes based on prior literature. Mutations were assumed to have GOF properties if previous literature has established their function, but other unknown mutations with oncogenic properties may be misclassified. Additionally, as this was a retrospective study, no link to causality can be definitively established. Although the cohort originated from a large, high volume, NCI‐designated comprehensive cancer center, its single‐center design may limit its generalizability. During the analysis for survival outcomes in single mutations, confounding variables were not able to be assessed due to the small size. It is also important to note that this study only included patients that underwent curative‐intent resection, which limits the interpretation of the findings to the setting of early resectable PDAC.

## 5 Conclusions

Pancreatic cancer is an aggressive malignancy with improving treatments but poor overall survival. The P53 tumor suppressor gene, commonly mutated in PDAC, may provide prognostic clues depending on mutation function. We found that patients with non‐GOF P53 mutations had a higher incidence of lymphovascular invasion and shorter overall and disease‐free survival as compared to both GOF and WT patients. This effect was most prominent with FOLFIRINOX‐exposed patients, and non‐GOF mutation status became a negative prognostic factor of survival.

## Conflicts of Interest

The authors declare no conflicts of interest.

## Synopsis

This retrospective cohort study evaluates the impact of P53 mutation subtypes on the survival outcomes of patients with pancreatic ductal adenocarcinoma who underwent curative‐intent resection. The study included 330 patients with next‐generation sequencing data. Patients with non‐gain‐of‐function (GOF) P53 mutations displayed higher rates of tumoral lymphovascular invasion compared to patients GOF or wild‐type P53 expression. Non‐GOF P53 mutations were associated with worse recurrence‐free and overall survival. These findings were strongly associated with FOLFIRINOX treatment, suggesting that pancreatic cancers with P53 non‐GOF mutation display an increased chemoresistance to FOLFIRINOX treatment.

## Supporting information


**Figure 1:** Kaplan‐Meier curve for overall survival (OS) for patients who received FOLFIRINOX by P53 mutation subtype.
**Figure 2:** Kaplan‐Meier curve for disease‐free survival (DFS) for patients who received FOLFIRINOX by P53 mutation subtype.
**Figure 3:** Kaplan‐Meier curve for overall survival (OS) for patients who received Gemcitabine‐based chemotherapy by P53 mutation subtype.
**Figure 4:** Kaplan‐Meier curve for disease‐free survival (DFS) for patients who received Gemcitabine‐based chemotherapy by P53 mutation subtype.
**Table 1:** Optimized multivariable Cox regression model of overall survival in patients with exposure to FOLFIRINOX therapy and curative‐intent resection of pancreatic ductal adenocarcinoma.
**Table 2:** Optimized multivariable Cox regression model of disease‐free survival in patients with exposure to FOLFIRINOX therapy and curative‐intent resection of pancreatic ductal adenocarcinoma.
**Table 3:** Optimized multivariable Cox regression model of overall survival in patients with exposure to Gemcitabine‐based therapy and curative‐intent resection of pancreatic ductal adenocarcinoma.

## Data Availability

The data that support the findings of this study are available on request from the corresponding author. The data are not publicly available due to privacy or ethical restrictions.
