# Supplementary material for: P53 Function Status Correlates With Overall Survival in Patients With Resected Pancreatic Cancer
Source: J Surg Oncol. 2025 Aug 7;132(5):935–44. doi: 10.1002/jso.70060 (PMC12501923; doi:10.1002/jso.70060)
Supplement: Supplementary file 1 — Figure 1: Kaplan‐Meier curve for overall survival (OS) for patients who received FOLFIRINOX by P53 mutation subtype. Figure 2: Kaplan‐Meier curve for disease‐free survival (DFS) for patients who received FOLFIRINOX by P53 mutation subtype. Figure 3: Kaplan‐Meier curve for overall survival (OS) for patients who received Gemcitabine‐based chemotherapy by P53 mutation subtype. Figure 4: Kaplan‐Meier curve for disease‐free survival (DFS) for patients who received Gemcitabine‐based chemotherapy by P53 mutation subtype. Table 1: Optimized multivariable Cox regression model of overall survival in patients with exposure to FOLFIRINOX therapy and curative‐intent resection of pancreatic ductal adenocarcinoma. Table 2: Optimized multivariable Cox regression model of disease‐free survival in patients with exposure to FOLFIRINOX therapy and curative‐intent resection of pancreatic ductal adenocarcinoma. Table 3: Optimized multivariable Cox regression model of overall survival in patients with exposure to Gemcitabine‐based therapy and curative‐intent resection of pancreatic ductal adenocarcinoma. [file JSO-132-935-s001.docx]

**P53 Function Status Correlates with Overall Survival in Patients with Resected Pancreatic Cancer**

Supplemental Figures

Figure 1. Kaplan-Meier curve for overall survival (OS) for patients who received FOLFIRINOX by P53 mutation subtype. The median overall OS for the cohort was 29.4 ± 1.9 months (OS for wild-type group - 44.1 ± 8.7 months, OS for GOF group - 37.4 ± 6.3 months, and OS for non-GOF group - 23.9 ± 3.3 months (P=0.008). GOF – gain-of-function.


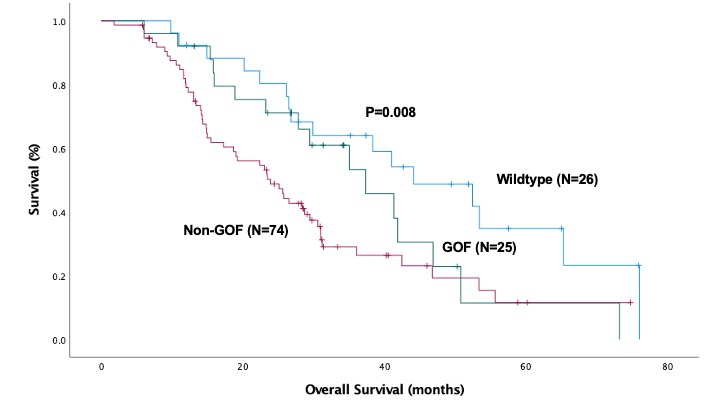


Figure 2. Kaplan-Meier curve for disease-free survival (DFS) for patients who received FOLFIRINOX by P53 mutation subtype. The median overall DFS for the cohort was 16.3 ± 1.3 months (DFS for wild-type group - 22.4 ± 4.2 months, DFS for GOF group - 18.3 ± 4.9 months, and DFS for non-GOF group - 12.7 ± 1.5 months (P=0.05). GOF – gain-of-function.


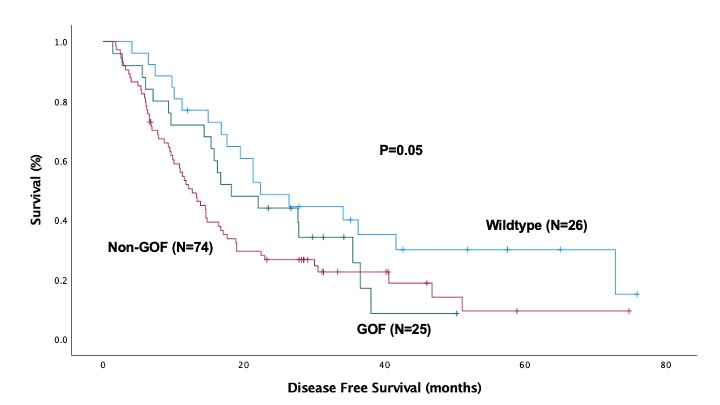


Figure 3. Kaplan-Meier curve for overall survival (OS) for patients who received Gemcitabine-based chemotherapy by P53 mutation subtype. The median overall OS for the cohort was 28.9 ± 2.7 months (OS for wild-type group – 27.4 ± 2.8 months, OS for GOF group - 35.0 ± 10.8 months, and OS for non-GOF group - 28.9 ± 4.2 months (P=0.55). GOF – gain-of-function.


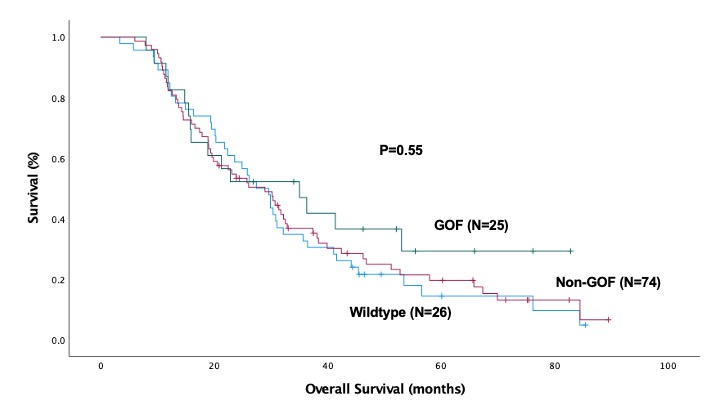


Figure 4. Kaplan-Meier curve for disease-free survival (DFS) for patients who received Gemcitabine-based chemotherapy by P53 mutation subtype. The median overall DFS for the cohort was 14.5 ± 1.3 months (DFS for wild-type group – 11.7 ± 1.5 months, DFS for GOF group - 16.9 ± 2.2 months, and DFS for non-GOF group - 14.6 ± 1.6 months (P=0.55). GOF – gain-of-function.


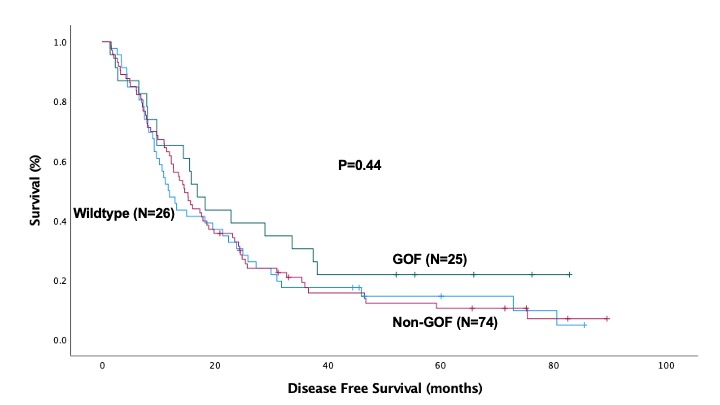


Supplemental Tables

Table 1. Optimized multivariable Cox regression model of overall survival in patients with exposure to FOLFIRINOX therapy and curative-intent resection of pancreatic ductal adenocarcinoma. GOF – gain-of-function, WT – wildtype, Sig – significance.

| **Covariate** | **Hazard ratios [95% CI]** | **Sig.** |
| --- | --- | --- |
| **Non-GOF vs WT/GOF** | 2.0 [1.2-3.2] | 0.008 |
| **Neoadjuvant chemotherapy** | 0.82 [0.49-1.4] | 0.45 |
| **Adjuvant chemotherapy** | 0.48 [0.23-1.0] | 0.058 |
| **T-stage I vs II** | 0.86 [0.45-1.5] | 0.62 |
| **T-stage I vs III** | 1.0 [0.51-2.0] | 0.95 |
| **T-stage I vs IV** | 13.4 [1.3-135.4] | 0.03 |
| **Positive margins** | 2.0 [1.1-3.6] | 0.028 |

Table 2. Optimized multivariable Cox regression model of disease-free survival in patients with exposure to FOLFIRINOX therapy and curative-intent resection of pancreatic ductal adenocarcinoma. GOF – gain-of-function, WT – wildtype, Sig – significance.

| **Covariate** | **Hazard ratios [95% CI]** | **Sig.** |
| --- | --- | --- |
| **Non-GOF vs WT/GOF** | 1.7 [1.1-2.6] | 0.016 |
| **Adjuvant chemotherapy** | 0.74 [0.39-1.4] | 0.35 |
| **T-stage I vs II** | 1.0 [0.59-1.7] | 0.99 |
| **T-stage I vs III** | 1.3 [0.68-2.4] | 0.45 |
| **T-stage I vs IV** | 6.8 [0.80-58.6] | 0.08 |
| **Positive margins** | 1.5 [0.86-2.7] | 0.15 |

Table 3. Optimized multivariable Cox regression model of overall survival in patients with exposure to Gemcitabine-based therapy and curative-intent resection of pancreatic ductal adenocarcinoma. GOF – gain-of-function, WT – wildtype, Sig – significance.

| **Covariate** | **Hazard ratios [95% CI]** | **Sig.** |
| --- | --- | --- |
| **Non-GOF vs WT/GOF** | 0.87 [0.59-1.3] | 0.47 |
| **Neoadjuvant chemotherapy** | 1.4 [0.85-2.2] | 0.20 |
| **Adjuvant chemotherapy** | 0.48 [0.21-1.1] | 0.09 |
| **T-stage I vs II** | 1.2 [0.66-2.3] | 0.50 |
| **T-stage I vs III** | 0.7 [0.38-1.4] | 0.32 |
| **Positive margins** | 3.6 [1.9-6.8] | <0.001 |
| **Lymphovascular invasion** | 2.3 [1.5-3.5] | <0.001 |
